# NEWBORN SCREENING FOR SEVERE COMBINED IMMUNODEFICIENCIES USING TRECS AND KRECS: SECOND PILOT STUDY IN BRAZIL

**DOI:** 10.1590/1984-0462/;2017;35;1;00013

**Published:** 2017

**Authors:** Marilia Pyles P. Kanegae, Lucila Akune Barreiros, Jusley Lira Sousa, Marco Antônio S. Brito, Edgar Borges de Oliveira, Lara Pereira Soares, Juliana Themudo L. Mazzucchelli, Débora Quiorato Fernandes, Sonia Marchezi Hadachi, Silvia Maia Holanda, Flavia Alice T. M. Guimarães, Maura Aparecida P. V. V. Boacnin, Marley Aparecida L. Pereira, Joaquina Maria C. Bueno, Anete Sevciovic Grumach, Regina Sumiko W. Di Gesu, Amélia Miyashiro N. dos Santos, Newton Bellesi, Beatriz T. Costa-Carvalho, Antonio Condino-Neto

**Affiliations:** a Departamento de Imunologia, Universidade de São Paulo (USP), São Paulo, SP, Brasil.; b Hospital Municipal Dr. José de Carvalho Florence, São José dos Campos, SP, Brasil.; c Departamento de Pediatria, Universidade Federal de São Paulo (UNIFESP), São Paulo, SP, Brasil.; d Hospital da Criança Santo Antonio, Porto Alegre, RS, Brasil.; e APAE-SP, São Paulo, SP, Brasil.; f Amparo Maternal, São Paulo, SP, Brasil.; g Hospital Regional da Asa Sul, Brasília, DF, Brasil.; h Hospital Geral de Carapicuíba, Carapicuíba, SP, Brasil.; i Hospital Municipal São Luiz Gonzaga, SP, Brasil.; j Ambulatório de Infecções de Repetição, Faculdade de Medicina do ABC, Santo André, SP, Brasil.; k Hospital da Criança Conceição, Porto Alegre, RS, Brasil.; l Clínica de Medicina Preventiva do Pará (CLIMEP), Belém, PA, Brasil.

**Keywords:** Severe combined immunodeficiency (SCID), Agammaglobulinemia, Newborn screening, Immunologic deficiency syndromes, Child

## Abstract

**Objective:**

To validate the quantification of T-cell receptor excision circles (TRECs) and kappa-deleting recombination excision circles (KRECs) by real-time polymerase chain reaction (qRT-PCR) for newborn screening of primary immunodeficiencies with defects in T and/or B cells in Brazil.

**Methods:**

Blood samples from newborns and controls were collected on filter paper. DNA was extracted and TRECs, and KRECs were quantified by a duplex real-time PCR. The cutoff values were determined by receiver operating characteristic curve analysis using SPSS software (IBM^®^, Armonk, NY, USA).

**Results:**

Around 6,881 samples from newborns were collected and TRECs and KRECs were quantified. The TRECs values ranged between 1 and 1,006 TRECs/µL, with mean and median of 160 and 139 TRECs/µL, respectively. Three samples from patients with severe combined immunodeficiency (SCID) showed TRECs below 4/µL and a patient with DiGeorge syndrome showed undetectable TRECs. KRECs values ranged from 10 to 1,097 KRECs/µL, with mean and median of 130 and 108 KRECs/µL. Four patients with agammaglobulinemia had results below 4 KRECs/µL. The cutoff values were 15 TRECs/µL and 14 KRECs/µL and were established according to the receiver operating characteristic curve analysis, with 100% sensitivity for SCID and agammaglobulinemia detection, respectively.

**Conclusions:**

Quantification of TRECs and KRECs was able to diagnose children with T- and/or B-cell lymphopenia in our study, which validated the technique in Brazil and enabled us to implement the newborn screening program for SCID and agammaglobulinemia.

## RESUMO

**Objetivo:**

Validar a quantificação de *T-cell receptor excision circles* (TRECs) e *kappa-deleting recombination circles* (KRECs) por reação em cadeia de polimerase (*polymerase chain reaction*, PCR) em tempo real (qRT-PCR), para triagem neonatal de imunodeficiências primárias que cursam com defeitos nas células T e/ou B no Brasil.

**Métodos:**

Amostras de sangue de recém-nascidos (RN) e controles foram coletadas em papel-filtro. O DNA foi extraído e os TRECs e KRECs foram quantificados por reação duplex de qRT-PCR. O valor de corte foi determinado pela análise de *Receiver Operating Characteristics Curve*, utilizando-se o programa *Statistical Package for the Social Sciences* (SSPS) (IBM^®^, Armonk, NY, EUA).

**Resultados:**

6.881 amostras de RN foram analisadas quanto à concentração de TRECs e KRECs. Os valores de TRECs variaram entre 1 e 1.006 TRECs/µL, com média e mediana de 160 e 139 TRECs/µL, respectivamente. Três amostras de pacientes diagnosticados com imunodeficiência grave combinada (*severe combined immunodeficiency*, SCID) apresentaram valores de TRECs abaixo de 4/µL e um paciente com Síndrome de DiGeorge apresentou TRECs indetectáveis. Os valores de KRECs encontraram-se entre 10 e 1.097 KRECs/µL, com média e mediana de 130 e 108 KRECs/µL, e quatro pacientes com diagnóstico de agamaglobulinemia tiveram resultados abaixo de 4 KRECs/µL. Os valores de corte encontrados foram 15 TRECs/µL e 14 KRECs/µL, e foram estabelecidos de acordo com a análise da *Receiver Operating Characteristics Curve*, com sensibilidade de 100% para detecção de SCID e agamaglobulinemia, respectivamente.

**Conclusões:**

A quantificação de TRECs e KRECs foi capaz de diagnosticar crianças com linfopenias T e/ou B em nosso estudo, validando a técnica e dando o primeiro passo para a implementação da triagem neonatal em grande escala no Brasil.

## INTRODUCTION

Severe combined immunodeficiency (SCID) is a heterogeneous group of diseases characterized by a low number or absence of T lymphocytes. A defect in the antibody production, which may be due to intrinsic defects of B lymphocytes or inappropriate activity of T cells, is usually present.^1^ Patients with SCID are healthy at birth; however, they develop bacterial, viral, or fungal infections in the first months of life and, if not properly treated, they will die before reaching one year of age.^2^ ^,^ ^3^ Curative therapy is the hematopoietic stem cell transplantation (HSCT) or, in some cases, gene therapy. The earlier HSCT is performed, the better the prognosis.^4^

Since 2008, newborn screening for SCID has been available in the United States of America. The methodology consists in quantifying T-cell receptor excision circles (TRECs), which are small markers produced during the development of T lymphocytes in the thymus. During the recombination of T-cell receptor genes, DNA segments are excised, forming small circles named TRECs, which can be amplified by polymerase chain reaction (PCR). Its quantity in the peripheral blood directly reflects thymic activity.^3^ ^,^ ^5^

Recently, Borte et al.^6^ described a multiplex assay capable of detecting TRECs and kappa-deleting excision circles (KRECs) in the same reaction for newborn screening of primary immunodeficiencies. KRECs and TRECs are similarly formed during the development of B lymphocytes and their quantification enables early diagnosis of defects in B cells, such as X-linked agammaglobulinemia (XLA) or autosomal recessive. These are diseases characterized by B-cell deficiency caused by mutations in genes that encode components of the B-cell receptor (BCR) or precursor thereof (pre-BCR) and lead to decreased serum immunoglobulin levels. The predominant defect is XLA, corresponding to 85% of cases. Carriers of this disease are susceptible to viral and bacterial infections, and the most common causes of infection are the encapsulated bacteria *Haemophilus influenzae* and *Streptococcus pneumonia*. The onset of symptoms in agammaglobulinemia occurs between three and six months of age, as maternal immunoglobulin levels fall. If there is no family history, the diagnosis is usually late.^7^ ^,^ ^8^ Early identification of carriers of agamaglobulinemias is very advantageous, as these children are prone to develop chronic and debilitating respiratory infections.^3^ ^,^ ^6^

A previous work carried out by our group, which was pioneer in the country, has validated the TRECs quantification methodology for neonatal screening for SCID.^9^ This study aimed at validating the quantification of TRECs and KRECs in the same reaction by quantitative real-time PCR (qRT-PCR) for neonatal screening of primary immunodeficiencies which occur with defects in T and/or B cells. This enabled to widen the range of diseases screened.

## METHOD

This cross-sectional study included samples from newborns (NB), whose parents/guardians agreed to participate in the research project. Blood samples were collected between September 2014 and July 2015 on filter paper from heel puncture in the NB, in compliance with current ethical norms (CAAE: 36364214.8.0000.5467). Samples from three hospitals of the metropolitan region of São Paulo (*Amparo Maternal*, *Hospital Geral de Carapicuíba*, and *Hospital Municipal São Luiz Gonzaga*), from a clinic in Belém (PA - CLIMEP), a hospital in São José dos Campos (SP - *Hospital Municipal Dr. José de Carvalho Florence*), and a hospital in Porto Alegre (RS - *Hospital Nossa Senhora da Conceição*) were collected and analyzed. Twenty-three samples from patients with suspected primary immunodeficiency (Table 1)^10^ were referred for investigation by pediatric immunologists/allergists across the country. As positive controls for test validation, samples from three patients previously diagnosed with SCID and four patients with agammaglobulinemia were used.

**Table 1:**
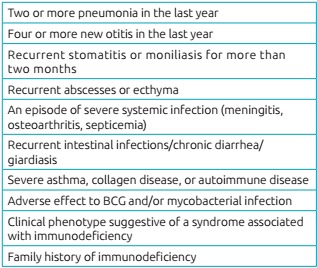
Ten signs of primary immunodeficiencies in infants.

Source: *Grupo Brasileiro de Imunodeficiência Primária* (BRAGID).

For PCR, we performed DNA elution from discs of 3.2 mm in diameter from the samples collected on filter paper.^11^ The DNA was then amplified in a duplex qRT-PCR for TRECs and KRECs in final volume of 20 uL, containing 10 uL of TaqMan Gene Expression Master Mix, 8 uL of DNA solution, 0.8 uL of 10 mg/mL bovine serum albumin, and primer and probes as described by Sottini et al*.* ^12^ The final concentrations of primers for TRECs and KRECs were 300 and 600nM, respectively, and probes were used at concentration of 200 nM. The amplification of beta-actin was performed on the same microplate, only for samples with TRECs and/or KRECs below the cutoff value, with primers and probe described by Baker et al.,^5^ at final concentrations of 250 and 150 nM, respectively. The concentrations of TREC, KREC, and beta-actin molecules (endogenous control) were calculated using one standard curve built by dilution of plasmids containing the specific sequences described by Sottini et al.^12^

To determine whether a sample was within the normal parameters, we used an initial cutoff value for TRECs and KRECs of 25 copies/uL, which was based on the study of Baker et al*.* ^5^ If the sample showed values below this concentration in the initial analysis, we repeated the whole process of extraction and amplification, along with the quantification of beta-actin. After this second step, if TRECs and/or KRECs remained below normal, with beta-actin above 8,000 copies/uL, patients were contacted and referred to pediatric immunologist/allergist for evaluation and confirmatory testing (immunophenotyping of lymphocytes and subpopulations).

The final data analysis was performed using descriptive statistics. We determined the non-Gaussian distribution of the sample by means of the Kolmogorov-Smirnov test, and data were presented according to median and interquartile values. Concentrations of TRECs and KRECs in full-term and preterm NBs were analyzed using the Mann-Whitney test in the software GraphPad Prism 5.0 (San Diego, CA, USA). Finally, we analyzed the receiver operating characteristic curve (area under the curve) to determine a final cutoff value, with 100% sensitivity for SCID and agammaglobulinemia detection, using the Statistical Package for Social Sciences (SPSS) (IBM^®^, Armonk, NY, USA).

## RESULTS

Around 6,881 samples from NBs were collected and analyzed for the concentration of TRECs and KRECs. Of the total sample, 1,853 (26.90%) were from CLIMEP (Belém/PA), 146 (2.10%) from the *Hospital Municipal Dr. José de Carvalho Florence* (São José dos Campos/SP), and 40 (0.58%) from the *Hospital Nossa Senhora da Conceição* (Porto Alegre/RS), and the remaining samples were from hospitals located in the metropolitan region of São Paulo.

The TRECs values ranged between 1 and 1,006 molecules/µL, with mean and median of 160 and 139 molecules µL, respectively. Three samples of patients diagnosed with SCID showed values of TRECs below 4/µL, and a patient with DiGeorge syndrome presented undetectable TRECs despite normal amplification of beta-actin (Table 1).^13^ KRECs values ranged between 10 and 1,097 molecules/µL, with mean and median of 130 and 108 molecules/µL. Four patients with agammaglobulinemia had results below 4 molecules/µL.

**Table 1:**
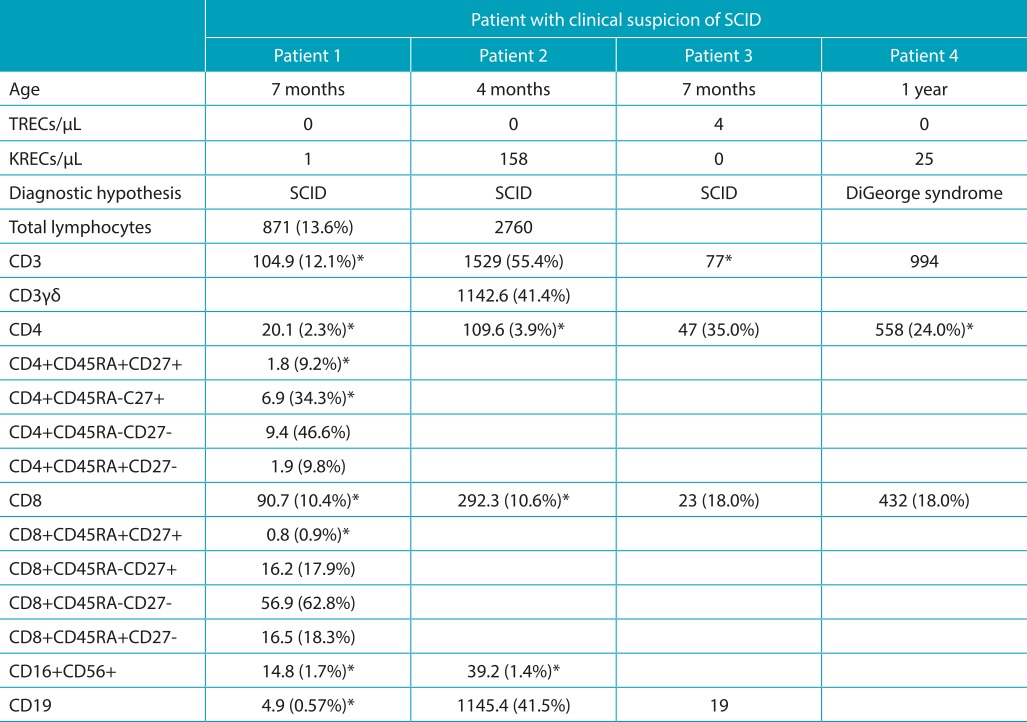
Immunophenotyping of lymphocytes from three patients with severe combined immunodeficiency and one with diagnosed DiGeorge syndrome who was referred as control in neonatal screening.

*Below p10 for Brazilian infants, according to Moraes-Pinto et al.^14^. KRECs: kappa-deleting recombination excision circles; TRECs: T-cell receptor excision circles; SCID: severe combined immunodeficiency.

**Figure 1:**
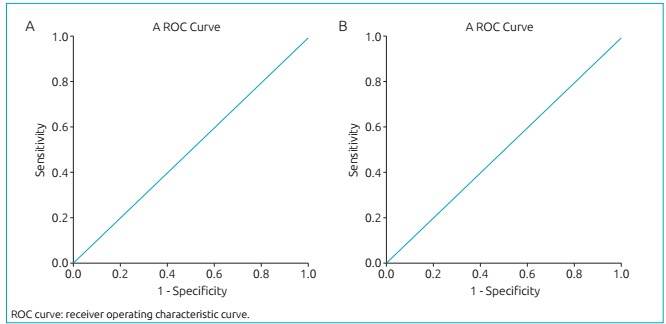
(A) Receiver operating characteristic curve for cutoff value of T-cell receptor excision circles of 15/µL, with area under the curve of 1.00. (B) Receiver operating characteristic curve for cutoff value of kappa-deleting recombination circles of 14/µL, with area under the curve of 1.00.

**Figure 2:**
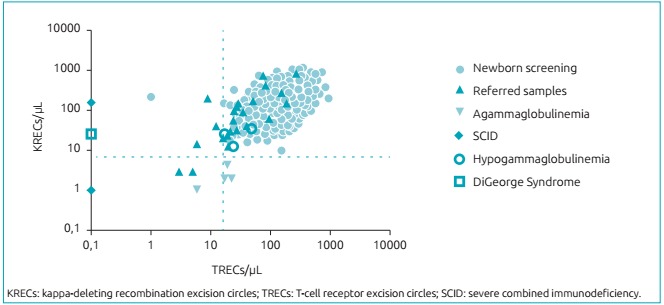
Concentration of T-cell receptor excision circles and kappa-deleting recombination circles of 6,881 samples of newborn screening and of 23 patients referred by pediatric immunologists/allergists for suspected primary immunodeficiency. T-cell receptor excision circles and kappa-deleting recombination excision circles were quantified by quantitative real-time polymerase chain reaction. Samples from patients already diagnosed with severe combined immunodeficiency, agammaglobulinemia, hypogammaglobulinemia, and DiGeorge syndrome were analyzed as controls for validation of the method. Dashed lines represent the cutoff values resulting from the receiver operating characteristic curve analysis.

The cutoff values were 15 TRECs/µL and 14 KRECs/µL, established according to the analysis of the receiver operating characteristic curve, with sensitivity of 100% for detection of SCID and agammaglobulinemia, respectively (Fig. 1). Samples with results below the cutoff values described earlier were subjected to new DNA extraction and were re-examined aggregating the beta-actin analysis for the extraction quality control. Only two samples from the newborn screening remained with low values of TRECs and/or KRECs, as shown in Fig. 2. These patients were contacted and referred to a pediatric immunologist/allergist, who was a collaborator to this work. The first patient, whose results were 1 TREC/µL and 211 KRECs/µL, died on the sixth day after birth as a result of a pleural effusion occurred at six months of pregnancy. As a consequence, parents received genetic counseling and a blood sample followed for genetic sequencing. The second patient (157 TRECs/µL and 10 KRECs/µL) attended the first medical appointment. No history related to primary immunodeficiencies or consanguinity was found on this occasion; however, this patient did not return to collect samples for specific tests. Twenty-three samples from patients with suspected primary immunodeficiencies (black triangles in Fig. 2) were referred by pediatric immunologists/allergists throughout Brazil. Of these, five resulted in TRECs and/or KRECs values below the cutoff. This result corroborated the clinical suspicion. One of these patients is carrier of trisomy 21 and presented values of 6 TRECs/µL and 14 KRECs/µL.

**Figure 3:**
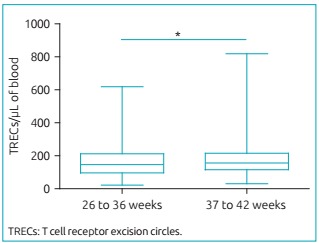
Concentration of TRECs/µL in 180 preterm newborns (26-36 weeks) and 1,368 full-term infants (37-42 weeks), quantified by real-time polymerase chain reaction. Preterm infants presented lower concentrations of T-cell receptor excision circles than those born at term (146 *versus* 156 TRECs/µL, respectively; *p<0.05, Mann-Whitney test). The concentration of T-cell receptor excision circles is represented in box plot showing median, 25th and 75th percentiles, and minimum and maximum.

A total of 1,548 samples from Belém (PA) were analyzed for gestational age and TRECs and KRECs values, because there are reports in the literature of preterm infants who have lower TRECs values,^13^ ^,^ ^15^ which would represent a false-positive risk. Fig. 3 shows that TRECs values were significantly lower in premature infants *(p*<0.05). They showed median of 146 TRECs/µL, whereas those born at term had median of 156 TRECs/µL. KRECs values did not vary according to gestational age.

## DISCUSSION

We analyzed 6,881 samples from different states of Brazil, along with positive controls of SCID and agammaglobulinemia, which validated the assay to quantify the concentration of TRECs and KRECs.

For the first 1,000 samples analyzed, cutoff values of 25 TRECs/µL and 25 KRECs/µL were initially applied, which resulted in a high repetition rate - 2.5% of the samples had concentrations below the cutoff values in a first analysis - compared to a previous work of our group,^9^ but still within the range reported by other authors (0.20-3.26%).^4^ One possibility for this high repetition rate was the use of a plasmid which was different from the previously employed - only contained the sequence of TRECs. Therefore, it was decided to analyze a larger number of samples along with positive controls of the diseases under investigation in order to establish an appropriate cutoff value. Thus, after quantification of almost 7,000 samples, receiver operating characteristic curve was analyzed, and cutoff values of 15 TRECs/µL and 14 KRECs/µL, with 100% sensitivity for detection of SCID and agammaglobulinemia, respectively, were found. These values are very close to those reported by Borte et al.^8^ (15 TRECs/uL and 10 KRECs/uL) and resulted in a repetition rate of 0.49% (34 samples), which was very close to the value found in our previous work.^9^ After the reanalysis of these 34 samples that were below the new cutoff values, only two samples (0.03%) remained altered. Confirmation of diagnosis of these two individuals was not possible owing to the absence of follow-up.

The repetition rate of the applied methodology, that is, the percentage of samples to be reanalyzed, is directly related to the restriction imposed by the cutoff value used. The lower the cutoff value, the smaller the number of samples to be resubmitted to all the procedure. Moreover, another important factor to be considered in determining cutoff values are the pathologies the newborn screening intends to detect. A recent review of newborn screening for SCID/T-cell lymphopenia showed that several other diseases that occur with T-cell lymphopenia can be detected by quantifying TRECs; however, some of these cases do not present undetectable or very low TRECs, as in classic SCID.^16^

Another interesting study quantified TRECs in carriers of 22q11 deletion and showed that cutoff variation leads to different success rates in the neonatal diagnosis of patients with this syndrome, and the lower the cutoff value, the lower the number of identified carriers.^17^ In our study, a patient with tetralogy of Fallot, hypocalcemia, and lymphopenia (CD3+:994/mm^3^) was referred to the *Laboratório de Imunologia Humana (ICB - USP)* owing to suspected DiGeorge syndrome, which was later confirmed by the detection of 22q11 deletion. TRECs were undetectable in this patient, being compatible with the number of T cells, which showed the usefulness of the assay in the detection of not only SCID, but also other diseases that progress with low number of T lymphocytes.

Similarly to TRECs, quantifying KRECs enables early detection of immunodeficiencies with defects in B-lymphocyte development. The methodology described in this study not only enables the identification of patients with agammaglobulinemia, but also assists in the classification of the type of SCID (T-B+; T-B-).^6^ With the advent of newborn screening for T- and B-cell lymphopenia, these children can be diagnosed soon after birth, as confirmed by the results of our study.

Of the three control patients with SCID in the study, two presented absent or very low number of TRECs and KRECs (SCID T-B-), and the third presented T-B+ phenotype, according to the qRT-PCR assay, and compatible with previous immunophenotyping of lymphocytes. As expected, the patient with DiGeorge syndrome presented normal value of KRECs.

Physicians who were collaborators of BRAGID network (Brazilian Group of Primary Immunodeficiencies, www.bragid.org.br) were responsible for the referral of 23 samples with suspected primary immunodeficiency. Of the total samples received, five had TRECs and/or KRECs values below the cutoff, which confirmed the clinical suspicion. One of these patients, who was carrier of trisomy 21, presented TRECs values below the cutoff, and consistent with previous American reports of newborn screening for SCID.^16^ Associated immunodeficiency was also found in this patient, warning physicians and caregivers about the additional precautions to be taken.

Analysis of samples from preterm and full-term infants confirmed data previously reported showing that preterm infants have lower TRECs values (146 *versus* 156 TRECs/µL).^13^ ^,^ ^15^ Therefore, we established that preterm infants with altered TRECs values at birth should undergo a second newborn screening for T-cell lymphopenia at an adjusted gestational age of 37 weeks. With regard to KRECs, the values were not statistically different between preterm and at term infants, as previously reported.^18^

Incidence of primary immunodeficiencies is unknown in Brazil. Many children are believed to die before the diagnosis is made, and therefore, as occurred in the United States of America before the neonatal screening, the incidence is probably underestimated.^19^ In Brazil, only patients with a family history of primary immunodeficiencies have the opportunity to benefit from early diagnosis. The onset of universal newborn screening for these conditions enables the early diagnosis and treatment for all children. The advent of TREC in newborn screening enabled to determine the incidence of SCID in the United States of America in 1:58.000.^16^ Taking into account the 2.9 million births/year in Brazil, we should diagnose approximately 50 patients per year. A recent research found a small number of cases of SCID diagnosed in Brazil and a high mortality rate.^20^ Incorporation of TRECs quantification in the neonatal screening in our country would benefit a significant number of patients with SCID.

SCID perfectly meets the criteria formulated by Wilson and Jungner to be included in newborn screening: absence of clinical signs and symptoms on physical examination, significant damage to the carrier if the disease is left untreated, available treatment, improved survival, and screening test available at a reasonable cost.^21^ Without proper diagnosis and treatment, children with SCID evolve to death in the first two years of life.^1^ Studies showed that the long-term survival, if NB patients undergo HSCT before three and a half months of age, is 94%. If transplantation occurs with active infections after this age, survival decreases to only 50%.^4^ Analyzing these numbers, we can conclude that early diagnosis can save the lives of children with SCID. The inclusion of KRECs quantification in the PCR at a minimal cost enables the diagnosis of primary B-cell immunodeficiencies and has proven capable of detecting patients with T-B-SCID and with agammaglobulinemia.

Currently, access to tests that quantify lymphocytes and their subpopulations is restricted to large centers and research laboratories. Quantification of TRECs and KRECs in dried blood samples on filter paper enables screening of samples coming from any geographic areas and the referral to specific tests, which save time and resources. In Brazil, there are no studies on the cost of patients with primary immunodeficiencies to the public health system. However, in the United States of America, studies have proven the cost-benefit of the inclusion of neonatal screening for T-cell lymphopenia, showing that the treatment of patients diagnosed before the onset of infections and consequent hospitalization cost up to four times less than of patients with late diagnosis.^22^

Finally, we conclude that the quantification of TRECs and KRECs detected children with T- and/or B-cell lymphopenia in our study. The technique is validated and can be widely employed in Brazil, without the need for additional sample collection, as the collection of the current neonatal screening test can be used in this assay. Therefore, we took the first step toward the inclusion of neonatal screening for T- and B-cell lymphopenia in our country, facilitating screening, early diagnosis, and treatment of patients with primary immunodeficiencies in Brazil.
